# Application of Mass Spectrometry Imaging in Uro-Oncology: Discovering Potential Biomarkers

**DOI:** 10.3390/life12030366

**Published:** 2022-03-03

**Authors:** Péter Czétány, Stefánia Gitta, András Balló, Alexandra Sulc, Gábor Máté, Árpád Szántó, László Márk

**Affiliations:** 1 National Human Reproduction Laboratory, 7624 Pécs, Hungary; czetany.peter@pte.hu (P.C.); stefania.gitta@aok.pte.hu (S.G.); ballo.andras@pte.hu (A.B.); alexandra.sulc@gmail.com (A.S.); magoaat@gmail.com (G.M.); szanto.arpad@pte.hu (Á.S.); 2 Urology Clinic, University of Pécs Clinical Centre, 7621 Pécs, Hungary; 3 Department of Biochemistry and Medical Chemistry, University of Pécs Medical School, 7624 Pécs, Hungary; 4 Pannon Reproduction Institute, 8300 Tapolca, Hungary; 5 MTA-PTE Human Reproduction Research Group, 7624 Pécs, Hungary

**Keywords:** urology, oncology, biomarker, mass spectrometry, renal cancer, bladder cancer, prostate cancer, testicular cancer

## Abstract

A growing need is emerging worldwide for new molecular markers which could enhance the accuracy of diagnostic and therapeutic methods for detecting urogenital cancers. Mass spectrometry imaging (MSI) is a very promising tool in this regard. In this review, we attempt to provide a subjective summary of the latest publications on potential biomarkers of renal, bladder, prostate, and testicular malignancies detected with MSI through the eyes of a clinical urologist.

## 1. Introduction

Urological malignancies form a large proportion of all cancers globally, their rising occurence and mortality mean a huge burden for health care systems worldwide [1]. Thus, it is essential to discover new biomarkers which could increase diagnostic accuracy and the efficacy of management in order to aid assessment and prognosis, increase recovery rate, and lower mortality.

Mass spectrometry imaging is a new and rapidly evolving technique that allows molecular signals to be evaluated directly from the surface of intact tissues. The name “imaging” was first used by the Caprioli group in 1997 [2]. Today, pharmaceutical companies are the largest users of this technique, using it to perform pharmacokinetic monitoring, pharmacotoxicology, and pharmacometabolomic analysis [3]. The method informs users about the molecular characteristics of a sample based on the mass-to-charge (*m/z*) ratio of molecules and provides an image of their spatial distribution. Imaging is an unlabeled technique that allows examination of the localization of up to hundreds of molecules within a tissue sample with a single measurement, and can later visualize them in 2D or even 3D. The technique can be used to perform unlabeled imaging of histological sections, cells, and cell cultures even at cellular resolutions. It can make peptides, proteins, and other metabolites visible, and is thus a “strong and unique tool for metabolome-mapping” [3]. The technique has such a high sensitivity that low femtomol and in some cases even attomol ranges can be reached when testing for proteins and peptides, which allows proper investigation of complex biochemical structures [4]. Another advantage is that the method is suitable for the detection of low molecular weight compounds and even lipids [3].

Although the MSI technique is extremely useful based on the above, there are very important steps in the method, such as tissue preparation. The tissue section to be examined should be placed on an indium tin oxide (ITO)-coated glass slide, which should then be coated as evenly as possible with a matrix corresponding to the analyte to be detected. The thickness, homogeneity, and crystallization of the sprayed matrix are critical to the resulting image. After the properly-prepared section is entered into the MS, the instrument uses a laser beam to make a mass spectrogram of each pixel of the selected sample, which are then summed. From the specific *m/z* values obtained in this way, the system first creates 2D images in which the color coding means different *m/z* values and the brightness is determined by the abundance of the given analyte. This is followed by another critical step in the measurement; the *m/z* values obtained must be identified and associated with a concrete molecule. The molecules can be identified based on their intact mass by matching to databases of known molecules; otherwise, tandem mass spectrometry measurement must be performed, which is limited and cumbersome in case of MSI. Finally, the resulting MS image should be compared with a histological image of the sample in order for anatomy structures to be easily identified [3].

Mass spectrometry imaging is an important analytical method for pharmaceutical companies, and is becoming increasingly important in clinical practice; it is a high-end, sensitive, and universally-applicable method that requires only small amounts of sample [4]. MSI has emerging significance in the pursuit of new disease biomarkers in urology [5]. The detection of new disease-specific biomarkers is important, and has been favored by the development of MS devices in recent years. Due to this, a relatively large number of biomarkers are already known among proteins. The advantage of the technique in this respect is that new biomarkers can be discovered only by comparing normal and abnormal tissue details in the sample, such that no prior knowledge or assumption is required. The detection of such biomarkers is important for the determination of abnormal and normal tissue boundaries, and by focusing on a particular molecule, potential drugs can be developed. Today, the medical use of MS includes the monitoring of molecular changes in normal and diseased organs, the localization of drug molecules in treated tissues, and the monitoring of any molecular changes that may occur as a result of any kind of treatment [6].

Despite the wide spectrum of urological biomarker research in the last decades, only a few have translated into clinical practice. MSI is an important resource for biomarker discovery; however, its application as a routine diagnostic tool faces difficulties as its techniqual requirements are expensive and its methodology requires special expertise. The few biomarkers validated in a clinical setting and approved for clinical use by the Food and Drug Administration (FDA) have mostly been discovered with immunological techniques (e.g., Nuclear Matrix Protein 22 (NMP22), Bladder Tumor Antigen (BTA) in bladder cancer) [7]. Nevertheless the candidate molecules discovered with MSI can be detected by other methods (e.g., immunoassays) in most cases (e.g., NPM22 was detected with MSI as well [8]), suggesting that MSI still has a promising role to play in this respect.

## 2. Genitourinary Malignancies

### 2.1. Kidney Tumors

Local recurrence after removal of a tumor is a major concern in all fields of surgery. Oppenheimer et al. [9] examined 34 samples from patients undergoing radical nephrectomies due to renal cell carcinoma and proved that the molecular characteristics of the neoplastic tissue can be detected beyond the surgical margin in the surrounding normal-appearing tissues with matrix-assisted laser desorption/ionization mass spectrometry (MALDI-MS). They observed, for example, the underexpression of proteins involved in the mitochondrial electron transport system. The significance of these findings is that the extent of the compromised tissue beyond the margin correlates with the aggressiveness of the tumor, and could thus be a prognostic marker.

Renal oncocytoma (RO) is a benign mass showing minimal growth in size, while chromophobe renocellular carcinoma (chRCC) is a neoplasia with true malignant potential. Standard care of patients with RO is partial nephrectomy; however, active surveillance may be a safe option as well [10,11,12], and in this regard surgical management could be overtreatment. In contrast, if left untreated chRCC progresses, potentially causing metastatic disease and threatening the life of the patient. The differentiation of the two can be difficult with conventional histological methods. Zhang et al. [13] applied desorption electrospray ionization mass spectrometry (DESI-MS) to analyse 81 samples of normal kidney tissue, RO, and RCC, and according to the authors this method is able to discriminate with 100% sensitivity (per patient) between RO and chRCC as well as between three subtypes of RCC (ccRCC, ppRCC, chRCC). The same research group detected higher levels of glutathione in the case of chRCC, while several cardiolipin-related species indicated RO.

Lu et al. [14] used MSI successfully (99% accuracy) to differentiate between ccRCC and chRCC; however, theirs was only a proof-of-concept study because of its relatively small sample size (14 ccRCC, 13 chRCC).

**Figure 1:**
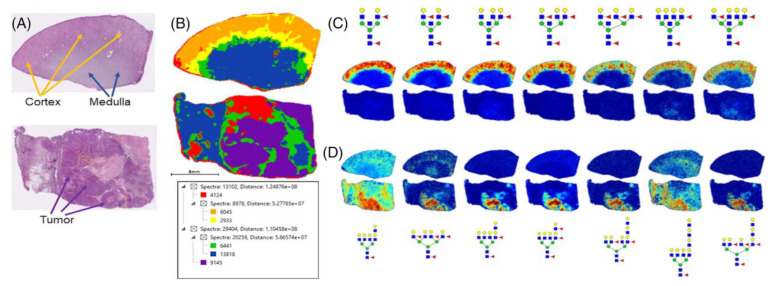
“Comparative N-glycan profiles of patient-matched normal and stage 4 ccRCC tissues. (**A**) H&E staining of patient-matched distal normal kidney (top) and stage 4 ccRCC (bottom) tissues. Tissue regions of cortex, medulla, and tumor are highlighted by arrows. (**B**) Segmentation analysis of 42 506 spectra and six nodes for 79 N-glycans in both tissues. (**C**) Normal kidney cortex region defined by glycans with bisecting GlcNAc residues and multiple fucosylated residues, which are absent in ccRCC tissues. (**D**) ccRCC-localized N-glycans that lack bisecting GlcNAc and show increased fucosylation and poly-LacNAc modification. Permission for reproduction obtained from John Wiley and Sons, Inc., Drake et al. [15].

Drake and his colleagues [15] examined the N-glycome of tumorous and non-tumorous renal tissues with MALDI–Fourier transform ion cyclotron resonance mass spectrometry (MALDI-FTICR MS). According to their results, the cortex and the medulla within the sample have a distinct characteristic glycan constitution; fucosylated bisecting GlcNAc N-glycans and high mannose structures are abudant in cortex tissues, while the predominant glycan structures of the medulla are biantennary and tri-antennary structures with a single fucose modification. They described a “unique glycomic structure pattern” localized at the interface between the two mentioned layers consisting of multifucosylated glycans that lack the bisecting GlcNAc. In RCC tissues, the bisecting GlcNAc glycans were minimally detectable or absent, while the most abudant structures characteristic to neoplastic tissue were tetraantennary glycans with multiple fucose residues, and the majority of the glycans detected above *m/z* = 2500 were tumor-associated (Figure 1). The authors correlated their previous transcriptomic gene array results with the study data. They investigated the expression of fucosyltransferases (FUT) and noticed the decreased levels of FUT3 and FUT6 in tumor tissues, while FUT11 was the only enzyme showing increased expression and could thus serve as a potential marker for RCC in the future. Glycans are complex molecules attached to the external surface of cells, and have vital functions in cell adhesion, signal transduction, and immune recognition. The basis of therapy for metastatic renal tumors is immunotherapy and inhibition of angiogenesis. There is an urgent need to find new therapeutic targets that provide better results and longer survival. Glycans, via their critical roles in cell function, might serve as the subject of research on this topic in the future.

### 2.2. Bladder Tumors

Bladder malignancies are divided into two main groups based on whether the tumor invades muscular tissue or not. Non-muscle-invasive neoplasias rarely progress into invasive disease and can be cured with transurethral resection (and adjuvant intravesical instillation of chemotherapeutic agent/BCG) without the need for systemic therapy, although they have a tendency to recur frequently, and thus patients require close follow-up. Muscle-invasive tumors progress rapidly, have high metastatic potential, and represent a life-threatening disease. There is an urgent need for molecular markers to diagnose recurrences early and to predict the clinical course of the disease. The current methods have low sensitivity for low grade cancer and in early stages [16,17].

Steurer et al. [18] applied MALDI-MS to tissue microarrays (TMA) of 697 bladder tumors. They observed two signals linked with recurrence in non-muscle-invasive cancers, one indicating a higher chance of progression to invasive tumor and one associated with longer tumor-specific survival in patients with invasive disease. Several of these signals could be identified as actual molecules, for example, a peptide from collagen alpha-1 accompanied both recurrence and transformation into invasive tumors. Two other signals linked to favorable phenotypes regarding prognosis were proven to be peptides from different subforms of keratin. These potential molecular markers could predict the risk of recurrence, could permit more precise determination of the interval of follow-up cystoscopies, avoiding unnecessary examinations. Signals of progression to muscle-invasive disease may be helpful to select patient candidates for early radical cystectomy, preventing progression into metastatic disease.

Carcinoma in situ (CIS) is a flat, non-invasive, and highly aggressive subtype of urothelial carcinoma, progressing into invasive disease in more than the half of cases [19]. The current gold standard method for assessment of the local extent of tumors as well as for grading is visual inspection of haematoxylin–eosin-stained tissue sections by a pathologist. This technique allows the possibility of interobserver variability, can lead to diagnostic failure in borderline cases, and may cause overtreatment. Particularly, differentiating between reactive urothelial atypia (RUA) caused by inflammation and CIS using conventional histopathological examination can be challenging, especially if the patient has previously been treated with intravesical instillation of Bacillus Calmette–Guérin (BCG), the standard treatment for CIS. After BCG treatment, reactive inflammatory processes in the mucosa are inevitable and can lead to a diagnostic dilemma when investigating potential recurrence/persistence of CIS.

**Figure 2:**
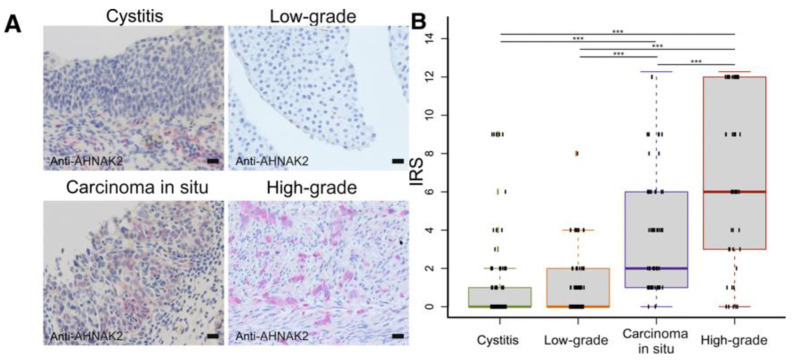
Evaluation of immune reactive score (IRS) for AHNAK2 in the second verification cohort: (**A**) Representative staining for AHNAK2 in each group (severe cystitis with reactive urothelial atypia (RUA), low-grade and invasive high-grade bladder cancer, and carcinoma in situ); (**B**) Boxplots display IRS for severe cystitis with RUA, low-grade and invasive high-grade bladder cancer, and carcinoma in situ; boxes represent the 25th and 75th percentiles, whiskers extend to the most extreme data points, and the median is shown as a horizontal line. Green indicates cystitis, orange indicates low-grade bladder cancer; blue indicates carcinoma in situ; and red indicates high-grade bladder cancer. n = 108 (**B**), severe cystitis with RUA; n = 84 (**B**), low-grade bladder cancer; n = 51 (**B**), invasive high-grade bladder cancer; n = 67 (**B**), carcinoma in situ. *** *p* ≤ 0.001. Scale bars = 20 μm (**A**). Permission for reproduction obtained from Elsevier Publishing, Witzke et al. [20].

Witzke and her colleagues [20] used liquid chromatography tandem mass spectrometry (LC-MS/MS)-based proteomic analysis to discover new biomarkers of bladder malignancies. They analyzed cystitis and high-grade urothelial carinoma samples. In a small cohort, they included low-grade urothelial carcinoma samples, and three potential biomarker proteins were identified: KRT6A, AHNAK2 and ASPN. KRT6A was highly expressed in cystitis and low-grade carcinoma cells, and was not present in high-grade carcinoma tissues. ASPN showed moderate expression in cystitis, moderate-to-high expression in low grade cancer, and high expression in high grade cancer. This protein was not previously correlated with bladder tumors. AHNAK2 was not expressed in cystitis, showed only marked expression in low-grade cancer, and had a high level in high-grade cancer. In a second verification step in a cohort including CIS samples, AHNAK2 was further analyzed. It showed high expression in CIS (Figure 2). The most significant differences were detected between RUA and CIS and between RUA and invasive high-grade cancer. A sensitivity of 97% was achieved in discrimination between cystitis and CIS, with a specificity of 69%, and between low-grade cancer and CIS with a specificity of 55%. In few cases in the RUA cohort, a higher immunreactivity was detected, and in one of the cases six months later low-grade cancer was diagnosed during the follow-up. Thus, the potential application of AHNAK2 as a prognostical marker appears promising.

### 2.3. Prostate Tumors

Diagnosis of prostate cancer (PCa) is based on histological examination of a sample gained by transrectal/transperineal needle biopsy. The result of conventional histological examination takes weeks, and it is impossible to resample if there is a strong clinical suspicion of cancer and the malignant foci are missed. Image-guided methods such as multiparametric magnetic resonance imaging (mp-MRI) and saturation biopsy can increase the sensitivity; however, neither provides immediate histological information.

Randall and her colleagues [21] used MALDI-MS and liquid extraction surface analysis mass spectrometry (LESA-MS) to examine specimens from radical prostatectomies. They observed several metabolic (especially lipidomic) changes related to tumorigenesis. Increased lipid intensity could be detected both in the malignant tissue and in the surrounding normal appearing tissue (“field cancerization” [22,23], similar to the results of the study by Oppenheimer et al. in case of kidney tumors). Higher intensities of certain lipids such as cardiolipins were associated with a higher Gleason grade during histopathological examination, suggesting that these molecules might be the indicators of more aggressive disease. This examination can be performed in less than two minutes according to the authors, and could thus provide in situ diagnosis and be utilized as a fast adjuvant diagnostic tool in the future.

**Figure 3:**
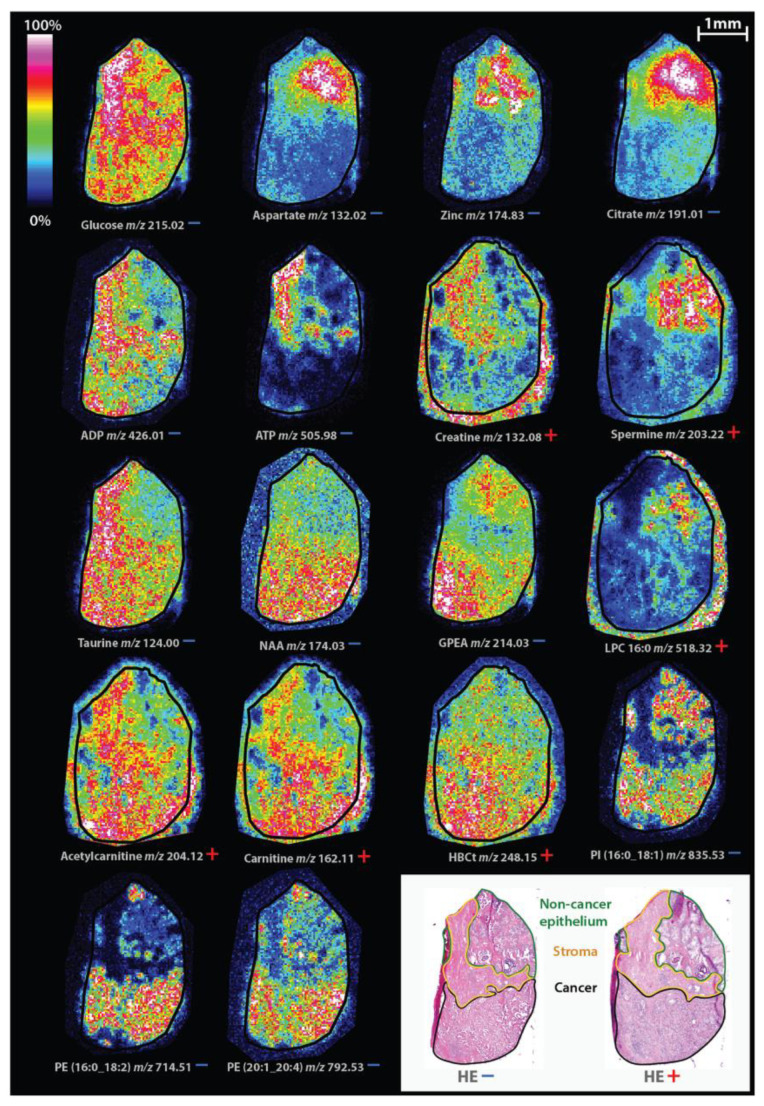
Spatial distribution of identified masses in both ion modes on consecutive tissue sections. Masses detected in positive and negative ion mode are indicated with a red plus and blue minus sign, respectively. For clarity, tissue edges are outlined with a black border. Note that the cancer region contains stroma finely mixed with cancer cells that could not be annotated separately. ADP = adenosine diphosphate, ATP = adenosine triphosphate, GPEA = glycerylphosphorylethanolamine, HBCt = hydroxybutyrylcarnitine, HE = hematoxylin and eosin, LPC = lysophosphatidylcholine, NAA = N-acetylaspartate, PE = phosphatidylethanolamine, PI = phosphatidylinositol. Permission for reproduction obtained from BioMed Central Ltd., London, Andersen et al. [24].

In a study by Andersen et al. [24], the metabolome and lipidome of non-tumorous prostatic epithelium, stroma, and cancer were compared by analyzing the tissue samples of fifteen patients who underwent radical prostatectomy with MALDI-TOF (time-of-flight) MSI. They used both the negative and positive ion detection modes to explore a wide spectrum of molecules. The prostatic stroma were examined separately, for the first time in an investigations of prostate samples using MSI. Measurements of stroma showed a distinct metabolic profile compared to both normal epithelium and malignant tissue, with high levels of taurine and adenosine phosphates. Most important markers differentiating between the epithelium and prostate cancer were citrate, zinc, and polyamine spermine, which showed decreased levels in neoplastic tissue. In the pathophysiology of prostate cancer, the role of these molecules’ altered concentrations is not fully known. In contrast to most neoplasia, which use glycolysis to meet increased energy requirements, the hallmark phenomenon of prostate cancer is slow glycolysis and intensified lipid metabolism [25]. These cells mainly use β-oxidation of fatty acids (FA) for energy production [26,27,28,29] and the increased rate of proliferation requires intensive phospholipid (PL) synthesis [25]. The research group detected higher levels of choline and glycerophosphoetanolamine, which are PLs constituting the cell membrane, in malignant tissue. The amount of another lipid metabolite, lysophosphocholine (LPC), was reduced in cancer cells, particularly of LPC (16:0), which in another study published by Goto et al. [30] was shown to be an independent predictor of biochemical recurrence after radical prostatectomy. β-oxidation requires the transport of FA through the mitochondrial membrane. The molecular mechanism responsible for this step is the so-called ’carnitine shuttle’, which attaches carnitine to FAs, making the mitochondrial membranes permeable for them via a transporter molecule. In this study, the components of this system (carnitine, acetylcarnitine, and hydroxybutyrylcarnitine) showed elevated levels in neoplastic cells (Figure 3). The molecules mentioned previously are candidates for clinical biomarkers of disease aggressivity, metastatic potential, recurrence, prognosis, and survival, in addition to their providing potential therapeutical targets in the future [25,26,27,28,29,30,31].

### 2.4. Testicular Tumors

Testicular cancer is the most frequently-diagnosed solid malignancy in young males. With modern platinum-based chemotherapeutic regimes it has become a highly curable disease, even in the advanced stages [32]. Seminolipids are glycolipids with an important role in spermatogenesis, and while they are abundant in normal testicular tissue, they are undetectable in malignant tissues [33,34].

**Figure 4:**
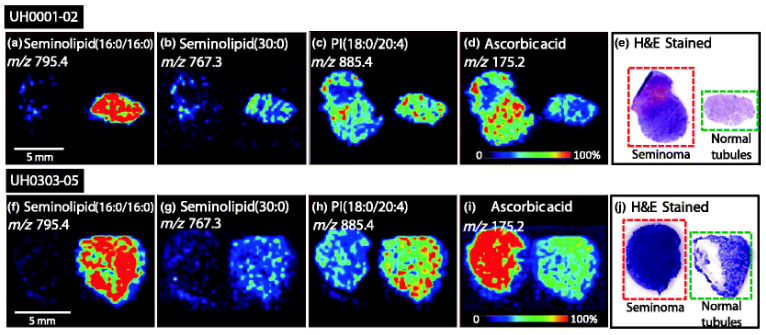
Negative ion mode tissue imaging of seminoma and adjacent normal tissue: (**a**) UH0001-02 ion image of *m/z* 795.4, seminolipid (16:0/16:0,); (**b**) UH0001-02 ion image of *m/z* 767.3, seminolipid (30:0); (**c**) UH0001-02 ion image of *m/z* 885.4, PI(18:0/20:4); (**d**) UH0001-02 ion image of *m/z* 175.2, ascorbic acid; (**e**) UH0001-02 H&E stained tissue sections of seminoma and normal tissue; (**f**) UH0303-05 ion image of *m/z* 795.4, seminolipid (16:0/16:0); (**g**) UH0303-05 ion image of *m/z* 767.3, seminolipid (30:0); (**h**) UH0303-05 ion image of *m/z* 885.4, PI(18:0/20:4); (**i**) UH0303-05 ion image of *m/z* 175.2, ascorbic acid; (**j**) UH0303-05 H&E-stained tissue sections of seminoma and normal tissue. Permission for reproduction obtained from the American Chemical Society, Masterson et al. [35].

In a study by Masterson et al. [35], their research group used DESI-MS to examine tumors histologically proven to be seminoma from fifteen patients who underwent inguinal semicastration along with the surrounding normal tissue. They found high intensities of seminolipids in normal tissues, whereas these molecules were completely undetectable in seminoma tissue, corresponding to the results of previous publications [34]. In contrast, they detected high concentrations of ascorbic acid in malignant tissues (Figure 4). This latter observation could have clinical significance in light of previous reports showing greater efficiacy of chemotherapy and radiotherapy in the presence of this substance [36,37].

In the case of imperative indication (bilateral synchronous tumor or metachronous contralateral tumor, young patient of fertile age before having children), testis-sparing surgery (TSS) can be recommended to the patient [38]. Other publications suggest that further criteria (single non-palpable mass, <2 cm, no marker elevation, normal hormone (LH, testosterone) levels, >60% of testicular volume can be conserved) should be fulfilled [39]. TSS means the resection of the tumor under surgical microscope and further sampling from the surgical margin. These samples are immediately transferred to the pathologist for frozen section examination (FSE). If a benign lesion or even malignant tissue with negative margins are reported, the testis can be spared with a close follow-up in the future. The method above may be a useful adjuvant diagnostic tool to augment the accuracy of FSE.

## 3. Conclusions

As can be seen from the studies mentioned above (Table 1), MSI can be a powerful tool in diagnostic evaluation, potentially improving the efficacy of conventional histopathological examinations, augmenting invasive diagnostical procedures (e.g., cystoscopy), and aiding in the assessment of disease recurrence risk, prognosis, and survival. These studies may indicate which biomarkers can optimize treatment and aid in the discovery of new therapeutical targets, eventually decreasing morbidity and mortality due to urological cancers; however, today the clinical translation of this technique remains in the future.

## Author Contributions

Writing—original draft preparation, P.C. and S.G.; writing—review and editing, P.C., S.G., A.B., A.S., G.M. and L.M.; supervision, Á.S. and L.M. All authors have read and agreed to the published version of the manuscript.

## Figures and Tables

**Table 1:** Summary of biomarker candidate molecules covered in this article.

Type of Malignancy	Molecule	Direction of Alteration, Potential Application	Reference
Kidney	Gluthatione	Increased level in chRCC, differentiating from RO	Zhang et al., 2020
	FUT11	Increased level	Drake et al., 2020
Bladder	Collagen alpha-1	Indicating recurrence and transformation to invasive malignancy	Steurer et al., 2014
	Two particular subforms of keratin (*m/z* = 850.4, *m/z* = 1104.4)	Assessing prognosis (accompanied with better outcome)	
	AHNAK2	Indicating high grade disease, particularly CIS	Witzke et al., 2019
Prostate	Citrate, zinc, polyamine spermine	Decreased levels	Andersen et al., 2021
	Choline, glycerophospho-etanolamine	Increased levels	
	Carnitine, acetylcarnitine, hydroxybutyrylcarnitine	Increased levels	
	LPC (16:0)	Decreased level, indicating biochemical recurrence after radical prostatectomy	Andersen et al., 2021, Goto et. al., 2015
Testis	Seminolipids	Decreased levels	Masterson et al., 2011
	Absorbic acid	Increased level	
